# Hunting for origins of migraine pain: cluster analysis of spontaneous and capsaicin-induced firing in meningeal trigeminal nerve fibers

**DOI:** 10.3389/fncel.2015.00287

**Published:** 2015-07-28

**Authors:** A. Zakharov, C. Vitale, E. Kilinc, K. Koroleva, D. Fayuk, I. Shelukhina, N. Naumenko, A. Skorinkin, R. Khazipov, R. Giniatullin

**Affiliations:** ^1^Laboratory of Neurobiology, Kazan Federal UniversityKazan, Russia; ^2^Department of Physiology, Kazan State Medical UniversityKazan, Russia; ^3^Department of Neurobiology, A.I. Virtanen Institute for Molecular Sciences, University of Eastern FinlandKuopio, Finland; ^4^Medical Faculty, Department of Physiology, Abant Izzet Baysal UniversityBolu, Turkey; ^5^Shemyakin-Ovchinnikov Institute of Bioorganic Chemistry RASMoscow, Russia; ^6^Kazan Institute of Biochemistry and BiophysicsKazan, Russia; ^7^INSERM U901/Aix Marseille UniversityMarseille, France

**Keywords:** pain, trigeminal nerve, spike, capsaicin, cluster analysis

## Abstract

Trigeminal nerves in meninges are implicated in generation of nociceptive firing underlying migraine pain. However, the neurochemical mechanisms of nociceptive firing in meningeal trigeminal nerves are little understood. In this study, using suction electrode recordings from peripheral branches of the trigeminal nerve in isolated rat meninges, we analyzed spontaneous and capsaicin-induced orthodromic spiking activity. In control, biphasic single spikes with variable amplitude and shapes were observed. Application of the transient receptor potential vanilloid 1 (TRPV1) agonist capsaicin to meninges dramatically increased firing whereas the amplitudes and shapes of spikes remained essentially unchanged. This effect was antagonized by the specific TRPV1 antagonist capsazepine. Using the clustering approach, several groups of uniform spikes (clusters) were identified. The clustering approach combined with capsaicin application allowed us to detect and to distinguish “responder” (65%) from “non-responder” clusters (35%). Notably, responders fired spikes at frequencies exceeding 10 Hz, high enough to provide postsynaptic temporal summation of excitation at brainstem and spinal cord level. Almost all spikes were suppressed by tetrodotoxin (TTX) suggesting an involvement of the TTX-sensitive sodium channels in nociceptive signaling at the peripheral branches of trigeminal neurons. Our analysis also identified transient (desensitizing) and long-lasting (slowly desensitizing) responses to the continuous application of capsaicin. Thus, the persistent activation of nociceptors in capsaicin-sensitive nerve fibers shown here may be involved in trigeminal pain signaling and plasticity along with the release of migraine-related neuropeptides from TRPV1 positive neurons. Furthermore, cluster analysis could be widely used to characterize the temporal and neurochemical profiles of other pain transducers likely implicated in migraine.

## Introduction

Migraine is the most common neurological disease, but the exact neurobiological mechanisms leading to migraine pain are not well understood. A widely held view suggests that migraine headaches originate from the activation of trigeminal nerve terminals in meninges followed by neuronal sensitization via release of migraine mediators (Goadsby, 2007; Levy, 2010) such as calcitonin gene related peptide (CGRP; Giniatullin et al., 2008). Meningeal tissues are innervated by peripheral branches of the trigeminal nerve consisting mainly of nociceptive non-myelinated C- and thin myelinated Aδ-fibers (Strassman and Levy, 2006). Recent studies have revealed innervation of meninges via V3 (mandibular branch of the trigeminal nerve) which sends nerve terminals through the *nervus spinosus* to tissues around the medial meningeal artery (MMA; Schueler et al., 2013, 2014).

The typical feature of nociceptive peptidergic C-fibers is an expression of capsaicin sensitive transient receptor potential vanilloid 1 (TRPV1) receptors (Julius and Basbaum, 2001; Moran et al., 2011), which serve as an integrator of various chemical, mechanical and temperature pain signals. Moreover, the activation of TRPV1 receptors by capsaicin is associated with release of the neuropeptide CGRP (Schueler et al., 2013; Shatillo et al., 2013) which is known as a main migraine mediator (Giniatullin et al., 2008). Paradoxically, agonists of TRPV1 receptors could also serve as analgesics, probably due to their ability to desensitize these receptors (Jara-Oseguera et al., 2008; Wong and Gavva, 2009; Premkumar, 2010). While well studied in the somata of cultured neurons, TRPV1 receptor mediated signaling and the functional role of TRPV1 receptor desensitization in the peripheral nerve terminals remains unknown.

Recently, extracellular electrode recordings from meningeal nerves activated by mechanical stimulation were used to study several key aspects of the generation and propagation of nociceptive signals (De Col et al., 2012; Uebner et al., 2014). However, mechanisms of chemical activation of meningeal nociceptors have been little studied so far.

In the current study, we used suction electrode recordings from peripheral branches of the trigeminal nerve in isolated rat meninges to explore the properties of ortodromically propagating spontaneous and capsaicin-induced spikes. This information was used to characterize the peripheral nociceptive coding at the origin of migraine pain. The cluster analysis of spontaneous and capsaicin-induced spikes allowed us to reveal a remarkable functional heterogeneity of meningeal nerve fibers, essential for shaping pain signals in migraine.

## Materials and Methods

### Preparation and Solutions

**Figure 1:**
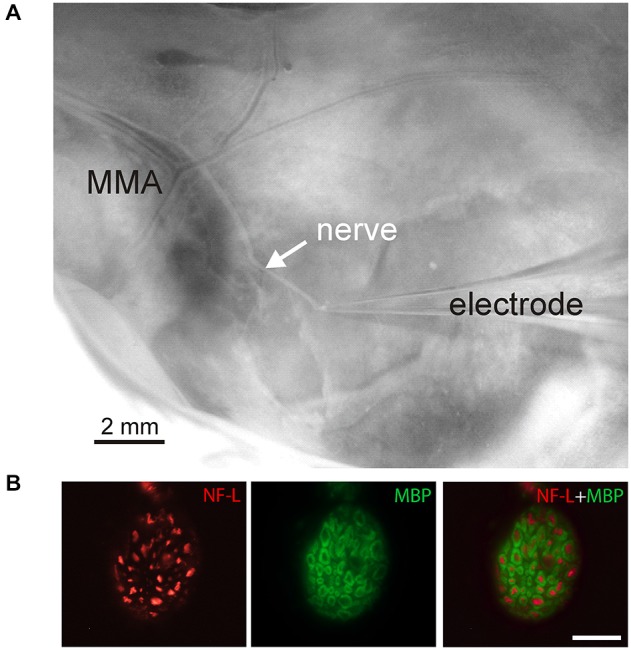
**Experimental setup for recordings from the *nervus spinosus*, branch of the trigeminal nerve in the hemiskull preparation. (A)** Photograph of the hemiskull preparation with preserved *nervus spinosus* (*nerve*) innervating a region of the medial meningeal artery (MMA). The nerve was cut and placed into the suction glass electrode. **(B)** Double immunolabeling of a cryostat cross-section of *nervus spinosus* with antibodies to a light component of neurofilament (NF-L, *red*) and to a myelin basic protein (MBP, *green*) showed the presence of myelinated NF-positive nerve fibers in the sample. Bar = 20 μm.

Experiments were carried out using isolated rat hemiskull preparations obtained from adult (P35–36) rats at room temperature (~20–22°C). All experiments were performed in accordance with the European Community Council Directive of September 22, 2010 (2010/63/EEC) and the experimental protocol was approved by the Animal Care and Use Committee of University of Eastern Finland. The hemiskull preparation (Figure 1) was placed into the experimental chamber (volume ~3 ml). The *nervus spinosus* (branch of the V3 branch of the trigeminal nerve) was carefully cleaned to remove the *dura mater* and prepared for placement into the recording system. This nerve innervates a region of the MMA, a likely place of origin of migraine pain and therefore represents a highly relevant model to study the mechanisms of migraine pain. Prior to recording, the isolated preparation was rinsed for ~20 min with Krebs solution. Krebs solution contained (in mM): NaCl 120, KCl 5, CaCl_2_ 2, MgCl_2_ 1, glucose 11, NaHPO_4_ 1, NaHCO_3_ 24, permanently gassed with 95% O_2_/5% CO_2_, starting usually 1 h before the experiment, pH was kept at pH 7.3–7.4. Capsaicin (Tocris, UK) was dissolved in Dimethyl sulfoxide (DMSO) and then to the final concentrations, 1, 2 or 10 μM in the bathing solution, and was bath applied via superfusion at ~7 ml/min driven by gravity. DMSO even in the highest (0.1% for dilution of 10 μM capsaicin) did not change the nociceptive firing (see “Results” Section for details).

For histochemical procedures *nervus spinosus* of adult P35–36 male rats was carefully dissected and fixed with 4% paraformaldehyde for 3–4 h. Tissue was intensively washed and stored overnight in 18% sucrose in phosphate buffered saline (PBS) for cryoprotection. Cryostat cross-sections (8 μm) of *nervus spinosus* were incubated for 1 h with PBS containing 5% bovine serum albumin (BSA; Sigma, Germany) and 0.5% Tween 20 for permeabilization and blockade of unspecific protein binding. A mixture of primary antibodies diluted in PBS containing 1% BSA was added to the samples to reach final concentrations as specified in Table 1. After overnight incubation at 4°C the sections were intensively washed with PBS and processed with appropriate combinations of secondary reagents (Table 1). Then, the samples were washed with PBS and coverslipped in an aqua mount medium (Sigma, Germany). The slides were evaluated by epifluorescence microscopy (Olympus IX70, Japan) using appropriate filter combinations.

**Table 1:** **Primary and secondary antibodies used**.

Primary reagents	Conjugate	Host	Dilution	Source
MBP		Rabbit	1:300	Abcam, UK
NF-L		Mouse	1:300	Invitrogen, USA
TRPV1		Rabbit	1:300	Alomone Labs, Israel
**Secondary reagents**	**Conjugate**	**Host**	**Dilution**	**Source**
Anti-mouse IgG	Biotin	Goat	1:200	Jackson Immunores. Lab. Inc., USA
Anti-rabbit IgG	Biotin	Donkey	1:200	Jackson Immunores. Lab. Inc., USA
Anti-mouse IgG	FITC	Rabbit	1:200	Chemicon, USA
Anti-rabbit IgG	Alexa Fluor 488	Donkey	1:200	Invitrogen, USA
Streptavidin	AMCA		1:200	Jackson Immunores. Lab. Inc., USA

For cytochemical staining of trigeminal ganglion cell cultures (3–5 days in culture) the same protocol was used with some modifications. Briefly, all procedures were carried out at room temperature, periods of incubation with 4% paraformaldehyde and primary antibodies were shortened to 20 min and 2 h, respectively.

Experiments with isolated trigeminal ganglion cells were carried out as described previously (Simonetti et al., 2006; Mazzuca et al., 2011). Briefly, neurons were plated on poly-L-lysine (0.2 mg/ml) coated cover slips and cultured for 1–2 days at 37°C in an atmosphere containing 5% CO_2_. The medium used was F12 supplemented with 10% fetal bovine serum with 50 ng/ml of nerve growth factor (NGF) added after cells were plated.

### Electrophysiology

The recording of spontaneous or capsaicin evoked spikes from the *nervus spinosus* was performed with a DAM80i amplifier (band pass 0.1–10 kHz, gain 10,000) using fire-polished glass microelectrodes with a tip diameter of ~150 μm, filled with Krebs solution. The *nervus spinosus* was cut at the entrance to the *dura mater*. By applying negative pressure via syringe, the distal part of the nerve stump was placed inside the recording microelectrode, this was followed by pressure relief. Stable baseline conditions were obtained after a recovery period of at least 15 min. Meningeal spikes were first recorded for 20 min as a control followed by 20 min of recording after capsaicin application. Each spike was visually inspected to prevent noise disturbance. All signals were digitized at 125 kHz using a data acquisition (DA) board NI PCI6221 (National Instruments, Austin, TX, USA), WinEDR software (Strathclyde University, UK) and stored on a PC and analyzed off-line with cluster analysis (MATLAB, Klustakwik).

Isolated TG neurons were recorded in whole-cell configuration in current clamp mode (*I* = 0). The cells were continuously superfused (at ~2 ml/min) with control solution containing (in mM): 152 NaCl, 5 KCl, 1 MgCl_2_, 2 CaCl_2_, 10 glucose, and 10 HEPES; pH was adjusted to 7.4 with NaOH and osmolarity was adjusted to 320 mOsM with sucrose. Patch pipettes had a resistance of 4–6 MΩ when filled with an intracellular solution containing (in mM): 135 CH_3_KSO_4_, 2 MgCl_2_, 2 NaATP, 0.5 NaGTP, 10 HEPES and 0.1 EGTA; osmolarity 300 mOsM, pH was adjusted to 7.2 with KOH. Capsaicin in these experiments was locally applied via the fast perfusion system (Rapid Solution Changer RSC-200, BioLogic Science Instruments, Grenoble, France), with solution exchange rate of ~20 ms. Responses to capsaicin (Sigma-Aldrich, Helsinki, Finland) were measured using a HEKA-10 amplifier and HEKA Patch Master software (HEKA Electronik, Germany).

### Cluster Spike Analysis

**Figure 2:**
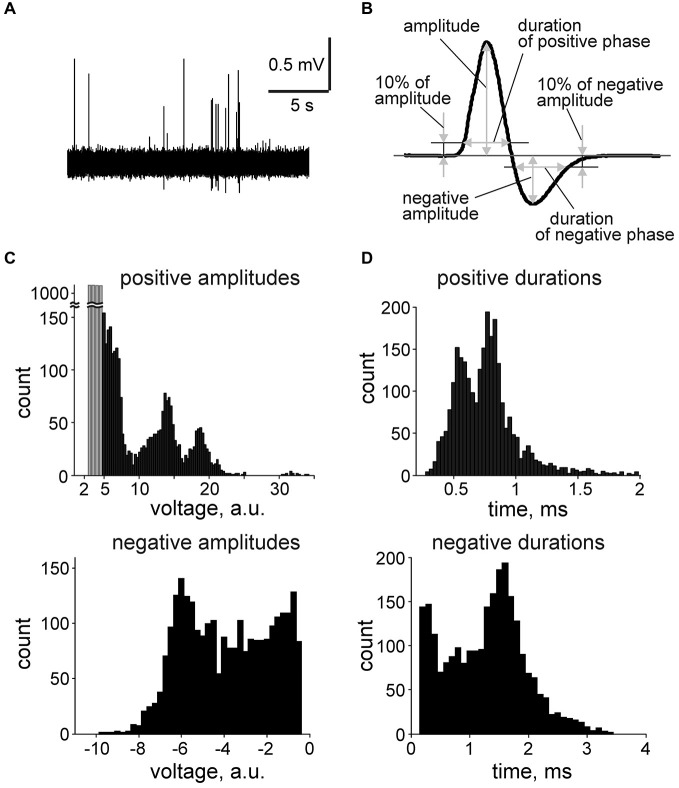
**Spontaneous multiple unit activity in** ***nervus spinosus***. **(A)** An example trace of spontaneous spiking activity recorded from the *nervus spinosus*. **(B)** Action potential parameters calculation in average spike. **(C)** Histograms showing the distribution of positive and negative spike amplitudes in one experiment built from *n* = 2627 spikes recorded during 15 min. Events below threshold (5 SD of baseline noise) are indicated in gray (*n* = 4100). **(D)** Histograms showing the distribution of positive and negative spike duration from the same experiment (*n* = 2627 spikes).

Original data were filtered at 100–9000 Hz (IIR, Chebyshev type II filter) for spike detection and cluster analysis. Baseline noise variance was calculated for each recording in 20 s long epochs with the minimal variance. The threshold for spike detection was set to five standard deviations (5SD) and spike amplitudes are presented in arbitrary units (a.u.) which represent peak spike amplitudes normalized to SD of each record. Spikes were analyzed in a time window of 2 ms before and 4 ms after the positive peak of the spike and the following spike parameters were calculated (Figure 2B): (1,2) positive and negative amplitudes; (3) the duration of the positive phase calculated at 10% positive amplitude threshold; (4) the duration of the negative phase measured at 10% negative amplitude threshold. Analysis of the digitized signal (filtering, detection, calculation of parameters) was performed using the MATLAB software (MathWorks, Natick, MA, USA). Clustering was performed using the KlustaKwik application (Kadir et al., 2014). In short, as the preliminary analysis showed an important role of the positive and negative spike amplitudes and durations in the unit identification, we plotted the positive amplitudes vs. duration or vs. the amplitude of the negative phase. Therefore, these spike properties were used as the input parameters for the KlustaKwik program. The clustering approach allowed us to divide the total flow of spikes into 3–10 separate clusters in each preparation (27–3153 spikes per cluster).

### Statistical Analysis

Statistical treatments of the data were made using Statistics toolbox (MATLAB). The two-side Wilcoxon rank sum test for paired samples was performed to assess parameter differences before and after capsaicin application in the same preparation whereas unpaired Wilcoxon test was used for unpaired samples. Significant difference was set at *p* < 0.05. Average values presented as mean ± SEM.

## Results

### Spontaneous and Capsaicin Induced Nerve Firing

Due to the placement of the cut peripheral part of the trigeminal nerve (*nervus spinosus*) inside the recording electrode (Figure 1A), we obtained exclusively the ortodromic traffic of peripherally generated action potentials (spikes). To examine a number of myelinated A-fibers in *nervus spinosus* we investigated two widely used neurochemical markers: neurofilaments (NF) and myelin basic protein (MBP; Lawson, 2002; Ivanusic et al., 2012; Bajaj et al., 2013; Flowerdew et al., 2013). Figure 1B presents the microscopic image of this nerve cross-section which contains ~50 myelinated A-fibers with the diameter in the range between 1 and 5 μm. Most of them were NF-immunopositive nerve fibers, especially, large sized ones. Due to small diameter and dense packing non-myelinated C-fibers of *nervus spinosus* were indistinguishable from each other by immunofluorescent microscopy that prevented their correct counting.

In control, spontaneous spikes generated in the *nervus spinosus* varied in their amplitudes and shapes (Figure 2A). The average shape of the typical meningeal spike consists of a short positive wave with a clear long lasting negative phase (Figure 2B). Multiple peak histograms of spike parameters for a single preparation (Figures 2C,D) suggested the presence of several groups of spikes. The positive wave duration of most spikes was in the range of 0.5–1 ms (Figure 2D). Such a short duration suggested that these signals were recorded from *single* fibers (multiple unit activity, MUA) rather than from groups of fibers giving longer lasting *compound* action potentials (De Col et al., 2012).

**Figure 3:**
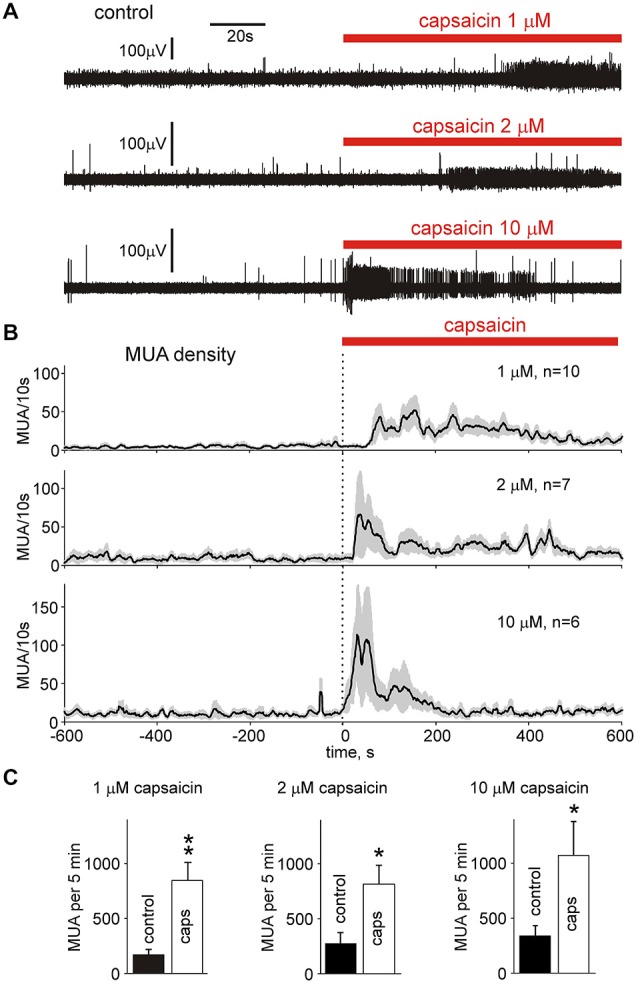
**Capsaicin increases multiple unit activity in** ***nervus spinosus.*** **(A)** Example of 200 s long recordings of multiple unit activity (MUA) in the *nervus spinosus* before and during bath-application of 1, 2 or 10 μM capsaicin. **(B)** MUA densities in the *nervus spinosus* before and during 1, 2 and 10 μM capsaicin application (mean ± SE; 10 s bin size; *n* = 10, 7 and 6, respectively). **(C)** Histograms quantifying the number of nociceptive spikes in control and after application of 1, 2 or 10 μM capsaicin (*n* = 10, 7 and 6, respectively). *p < 0.05, **p < 0.01.

Next, in order to characterize the nociceptive profile of meningeal afferent terminals, we tested the action of the “classical” algogen capsaicin, operating via TRPV1 receptors mainly expressed in peptidergic nociceptive C-fibers (Julius and Basbaum, 2001). Figure 3A shows non-clustered data from individual experiments where a large increase in MUA induced by an application of 1, 2 and 10 μM capsaicin was observed. The overall effect of 1 μM capsaicin on MUA frequency during 5 min of recording was 693 ± 149% increase over control (Figure 3B, top, Figure 3C, *n* = 10, *p* < 0.01), 673 ± 323% enhancement (Figure 3B, middle, Figure 3C, *n* = 7, *p* < 0.05) by 2 μM capsaicin whereas with 10 μM capsaicin the total increase was 527 ± 233% (Figure 3B, bottom, Figure 3C, *n* = 6, *p* < 0.05). Unlike time profile, the maximal effect of all concentrations of capsaicin was similar (*p* > 0.05 for all combinations by unpaired Wilcoxon test). DMSO used as vehicle in highest concentration of 0.1% did not change significantly the firing. Thus, the number of spikes after 0.1% DMSO application was 79 ± 15% of control (*p* > 0.05; *n* = 3) indicating the vehicle did not contribute to increased nociceptive firing during capsaicin applications.

**Figure 4:**
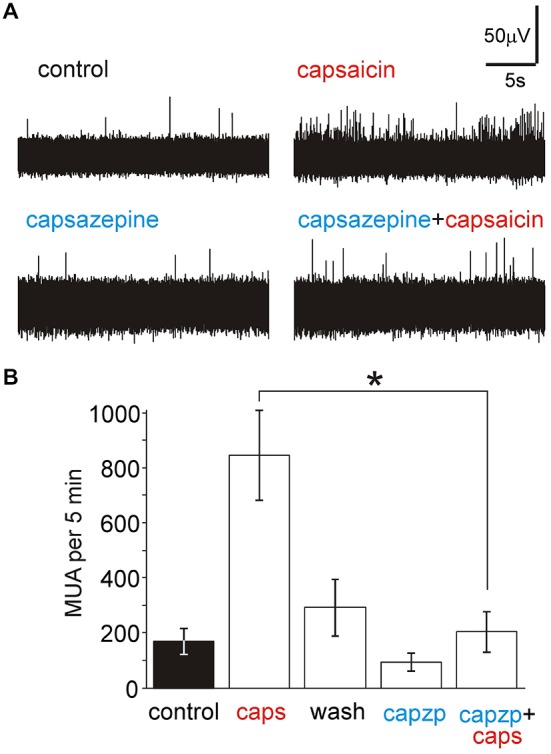
**Capsazepine prevents the activation of nociceptive firing by capsaicin in** ***nervus spinosus.*** **(A)** Example traces of the multiple unit activity stimulated by 1 μM capsaicin in control (top) and in the presence of 50 μM capsazepine (bottom). **(B)** Histograms showing the average effect of 1 μM capsaicin (*n* = 10), washout of the agonist (*n* = 10), effect of 50 μM capsazepine (*n* = 5) and action of 1 μM capsaicin in the presence of 50 μM capsazepine (*n* = 5) on the number of nociceptive spikes during 5 min of recordings. *p < 0.05.

In order to test the specific interaction of capsaicin with TRPV1 receptors we used the specific antagonist of TRPV1 receptors capsazepine. Figures 4A,B shows that the reversible action of 1 μM capsaicin (*n* = 10) was significantly reduced in the presence of 50 μM capsazepine (*n* = 5, *p* < 0.05 by unpaired Wilcoxon test), suggesting involvement of TRPV1 receptors.

Spontaneous and capsaicin-evoked spikes were strongly suppressed in the presence of the sodium channel blocker tetrodotoxin (TTX, 1 μM; *n* = 4, *p* < 0.05). In three out four experiments only 0–2 spikes were observed in the interval 8–10 min after TTX application compared to 50–80 spikes in the similar interval in control. This almost complete suppression indicated that these spikes were generated by TTX-sensitive subunits of sodium channels.

Figure 3B shows that the latent period of the capsaicin effect was dependent on concentration (probably due to different diffusion rate to individual fibers). However, enhanced activity maintained for at least 5–6 min (Figure 3B). Such long-lasting firing in meninges, in contrast to fast desensitizing responses to capsaicin in cell cultures (Joseph et al., 2013) could be explained by: (i) the asynchronous responses of different fibers; or (ii) non-desensitizing properties of capsaicin-sensitive TRPV1 receptors in this *ex vivo* preparation. Thus, we tested these possibilities using cluster analysis.

### Clustering of Spikes Revealed Responding and Non-Responding to Capsaicin Fibers

Clustering analysis by using KlustaKwik method (Kadir et al., 2014) was performed to detect distinct contributors to spontaneous and evoked firing of single fibers in order to understand the functional organization and diverse chemical sensitivity of individual fibers within the same meningeal nerve. Amplitudes and durations were found to be the most efficient parameters for the clustering of spikes in meninges.

The cluster analysis was based on detection of the most compact group of spikes distributed (mixture-of-gaussians) in the parameter space. The degree of “compactness” of the group was automatically calculated on the base of distances to each spike from the center of the group. Notably, the automatic clustering provided by the KlustaKwik method identifies (separates) different group of spikes even when they are different from others just only by one parameter (Kadir et al., 2014).

**Figure 5:**
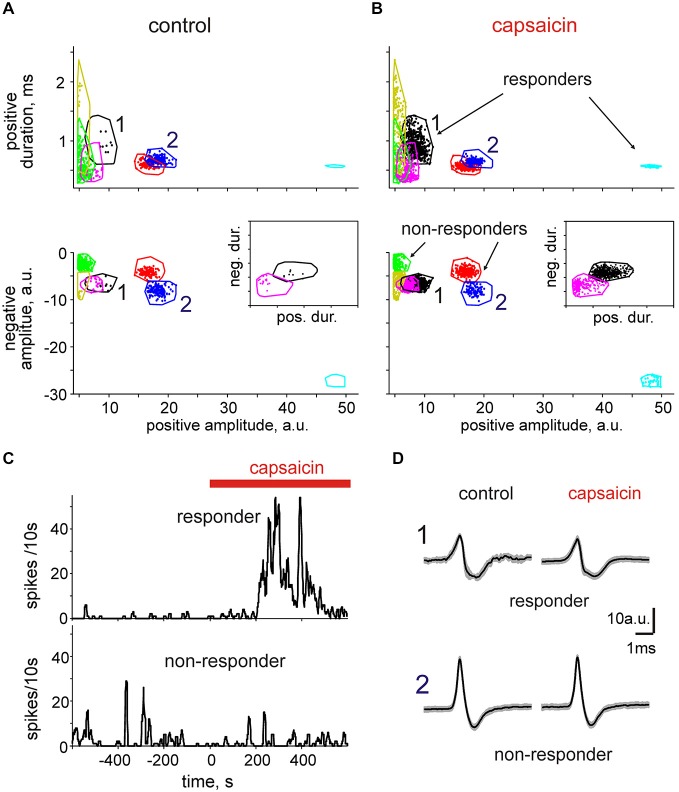
**Cluster analysis of spikes in** ***nervus spinosus***. **(A)** A typical presentation of spike clusters in 2D in control (spontaneous activity). Positive spike amplitudes are plotted vs. positive spike durations (top) and vs. the negative spike amplitudes (bottom). Each individual dot corresponds to a single spike. Color contours outline individual spike clusters separated on the basis of KlustaKwik methods. 1 and 2 indicate responder and non-responder clusters, respectively. **(B)** Spike clusters from the same predation as shown on panel **(A)** in the presence of 2 μM capsaicin. Notice that some units (black and magenta) increased firing during application of capsaicin (responders) whereas others (blue and green) were insensitive to capsaicin (non-responders). Inset indicates the isolated presentation of two clusters (magenta and black) when the negative duration was plotted vs. positive duration which were less distinguishable in other projections. **(C)** Time course of spike frequency of the units 1 (responder) and 2 (non-responder) before and after addition of capsaicin. **(D)** Average spike shapes for the clusters 1 and 2 (mean ± SD; responder: *n* = 9 in control, *n* = 475 in capsaicin; non-responder: *n* = 160 in control, *n* = 97 in capsaicin).

By plotting such parameters as the positive amplitude vs. positive duration or the positive amplitudes vs. negative amplitudes, we were able to distinguish individual clusters grouped from the total collection of spikes. Notably, the borders between clusters were most efficiently seen after applying several of these projections (Figure 5A). Thus, adding the third projection helped us further clarify the boarders between otherwise overlapping clusters. This is illustrated in the inset to the Figures 5A,B when the duration of the negative phase was plotted vs. the duration of the positive phase making it clear that overlapping clusters (*magenta and black*) in the projection “negative amplitude-positive amplitude” actually represent two different clusters.

Application of capsaicin was additionally useful in revealing those clusters (Figures 5A,B, cyan) which were completely silent in control. Figures 5A,B show that in the same meningeal nerve we were able to reveal six separate clusters, three of which were sensitive to capsaicin. Thus, one cluster marked as “1” with a low positive wave amplitude and a large long lasting negative component (Figures 5C,D upper) showed the strongest response to 2 μM capsaicin (240-fold increase in the mean frequency and 2700-times increase in the maximum frequency in 5 min interval). Interestingly, the other cluster 2 in the same nerve (Figures 5C,D, bottom) weakly (1.5-fold) reduced mean frequency in response to capsaicin.

Our approach revealed not only the presence of several clusters contributing to spontaneous activity of meningeal nerves but also the variable sensitivity (“responders” vs. “non-responders”) of clusters to the action of the algogen, capsaicin. Then we take an advantage from the separate monitoring of non-responders in order to test whether they remain unchanged during capsaicin application. In fact, we found a significant reduction in nociceptive firing of non-responders after application of 1 μM (67 ± 15% of control, *n* = 11, *p* < 0.05) or 2 μM capsaicin (59 ± 15% of control, *n* = 12, *p* < 0.001) although this effect was not visible with high 10 μM concentration of capsaicin (94 ± 15% of control, *n* = 14, *p* > 0.05). Although the detailed analysis of this phenomenon requires additional experiments our working hypothesis is that this reduction is mediated by capsaicin-induced release of extracellular messengers inhibiting neighboring fibers (non-responders) via the volume transmission mechanisms.

In general, from 23 experiments, 74 of 114 clusters (65%) showed high sensitivity (>2-times increase of mean frequency) to capsaicin. Interestingly, this value matches the proportion of isolated rat trigeminal neurons responding to capsaicin (60%, Simonetti et al., 2006).

### Capsaicin Action on Isolated Sensory Neurons

In order to quantify the parameters of capsaicin-induced firing in a single trigeminal neuron we performed recordings of spiking activity from isolated cultured trigeminal neurons in current clamp mode. One of the aims of this test was to quantify the average and *maximal* drug-induced firing frequency (in other words, the shortest interspike intervals) of single neurons. The measurement of firing activity of a single trigeminal neuron was important to link these data with the firing of single nerve fibers in the hemiskull preparation. The second aim was to explore the rate of desensitization of TRPV1 receptors in single neurons induced by paired capsaicin applications using a protocol analogous to Por et al. (2012).

**Figure 6:**
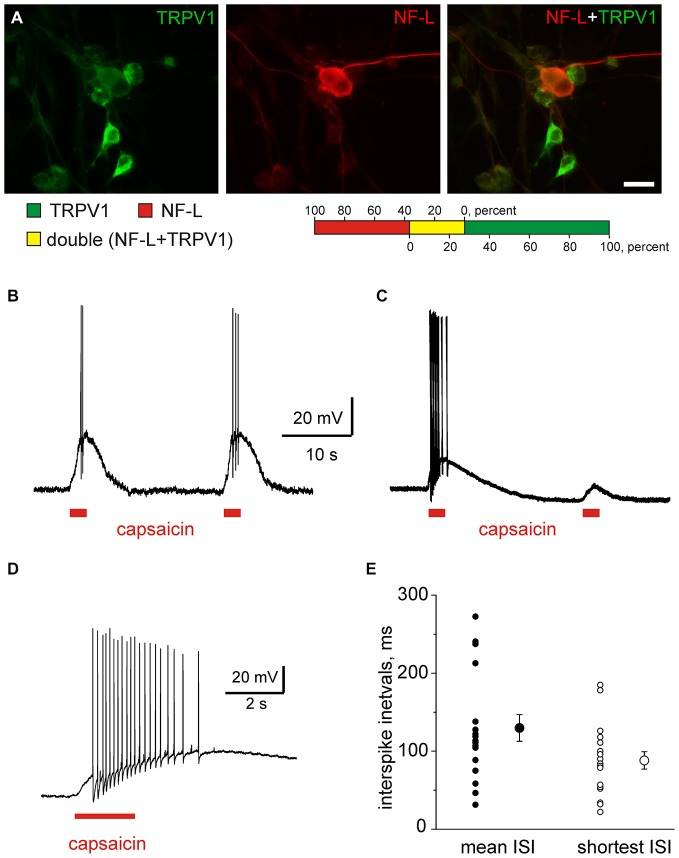
**Capsaicin induced firing in isolated trigeminal neurons. (A)** Double immunolabeling of cultured trigeminal ganglion neurons with antibodies to a capsaicin receptor transient receptor potential vanilloid 1 (TRPV1; *green*) and to a light component of neurofilament (NF-L, *red*). The picture indicates that the majority of TRPV1-expressed trigeminal neurons were NF-L immunonegative. Calibration bar = 20 μm. Color bar indicates percentage of TRPV1 and NF-L positive neurons and respective overlap of labeling. **(B**,**C)** Example traces of capsaicin (1 μM, 2 s pulses with 20 s intervals) with stable **(B)** and reduced amplitudes **(C)**. **(D)** Multiple firing induced by 1 μM capsaicin in an individual neuron. **(E)** Analysis of firing showing average and shortest intervals in individual neurons. Notice that three neurons generated firing with 20–30 ms intervals.

In our previous study, it was shown that 60% of cultured trigeminal ganglion neurons express TRPV1 receptor (Simonetti et al., 2006). In order to investigate whether myelinated fibers in the trigeminal nerve respond to capsaicin we evaluated the co-expression of TRPV1 receptors with the marker of myelinated fibers in isolated trigeminal neurons. For this aim we used double immunocytochemistry with antibodies to TRPV1 (*green*) and to a light component of neurofilaments (NF-L, *red*). Our data shown in Figure 6A suggest that among 153 NF-L positive neurons 56 (37%) also express TRPV1 markers. Among 174 TRPV1 positive neurons only 48 (28%) also express NF-L consistent with the view that TRPV1 receptors are mainly expressed in non-myelinated peptidergic neurons (Julius and Basbaum, 2001). Our data suggest that myelinated fibers shown in Figure 1B can contribute up to 1/3 of clusters activated by capsaicin.

Next, using patch clamp recordings we found that all trigeminal neurons could be divided into three groups. About half (47%, in total, *n* = 198 neurons) of all trigeminal neurons did not respond to 200 nM or 1 μM capsaicin. In some of responding neurons (*n* = 40) we applied paired pulse protocol (interval 20 s) and from those 52% (*n* = 21 neurons) responded to the second application of capsaicin with >15% reduced receptor potentials whereas the others generated responses with essentially the same amplitudes (compare A vs. B in Figure 6). Such variable kinetics of TRPV1 responses is consistent with the co-existence of several pools of TRPV1 receptors with distinct rate of desensitization determined by lipid environment and coupling to caveolins (Storti et al., 2015). These findings indicate an intrinsic heterogeneity of TRPV1 mediated responses, in particular in the sensitivity to capsaicin and the rate of desensitization (Storti et al., 2015). Data obtained from single neurons also predict variable persistence of capsaicin responses in trigeminal nerves which are present below.

Out of 105 trigeminal neurons responding to capsaicin, 25% (*n* = 26 neurons) generated multiple firing (Figures 6A–C). Interspike intervals for these firing neurons varied in a broad spectrum, from 20–2000 ms (Figure 6D). Figure 6D shows mean interspike intervals (corresponding to the range of 0.5÷50 Hz) and the shortest intervals in three neurones in Figure 6D were about 20–30 ms. This shortest interval was used as an indicator of maximal single fiber activity in the analysis of multiple firing activity of *nervus spinosus* (autocorrelation function, see below).

### Analysis of Multiple Firing Activity in Meningeal Nerve

**Figure 7:**
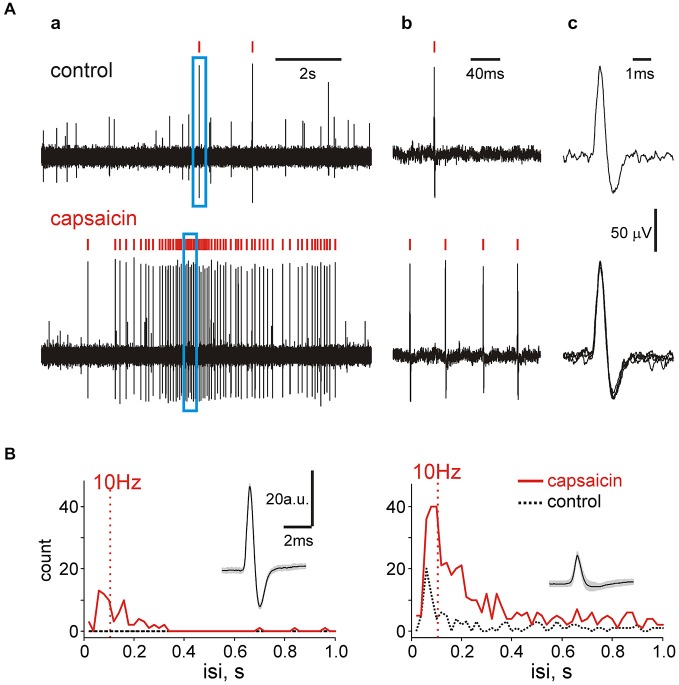
**Spectral analysis of single unit activity. (A)** Examples of spikes within the same cluster in two different time windows before and after capsaicin application. The right trace shows the shape of spikes belonging to the same unit. Red ticks above the trace in **(*a*)** indicate spikes of the same cluster whereas the blue frame—the part of the trace shown on the right **(*b*)** by higher temporal resolution. Notice that multiple firing in this small time window did not change the spike shape **(*c*)** inducing that they all belong to the same cluster. **(B)** The spectral analysis of spiking activity showing an increase in number of frequent spikes within two clusters (one with relatively high ongoing activity (right) and another one essentially silent prior to capsaicin application (left).

Nociceptive coding suggests more pain during high frequency spiking activity (Zhang et al., 2004). Our clustering approach suggests an attractive possibility to measure mean and maximal firing rates for individual sensory fibers in the trigeminal nerve which are critical for nociceptive discrimination at the level of brainstem. Therefore, we analyzed interspike intervals of such events in control and in the presence of capsaicin. Figure 7Aa shows a fragment of recording when only two large size spikes (marked by red ticks) appeared in control (top) but multiple firing was observed after capsaicin application (bottom). In the selected frame of 200 ms (blue frame in Figure 7Aa) there was just one spike in control conditions (Figure 7Ab) but after exposure to 10 μM capsaicin the number of similarly shaped spikes (suggesting their origin from the same fiber, see Figure 7Ac) was increased in this short interval up to 4. In order to provide a more detailed analysis of spiking activity, next we used the spectral analysis showing the predominant (and maximal) firing rates. Figure 7B shows data for two spike clusters in control and after capsaicin application. In both cases the occurrence of spikes following with a frequency exceeding 10 Hz in the presence of capsaicin was largely increased. This likely provides conditions for temporal summation of postsynaptic currents largely mediated by N-methyl-d-aspartate (NMDA) receptors at synapses between the nociceptive afferents and spinal dorsal horn neurons (Tong and MacDermott, 2014). Notably, 35/114 of capsaicin-responding clusters (31% of all clusters) showed firing frequency exceeding 10 Hz. Thus, within the same clusters the probability of firing at frequencies exceeding those needed for temporal summation at the brainstem and spinal cord level was largely increased.

### Time Profile of Capsaicin Action in Different Clusters

**Figure 8:**
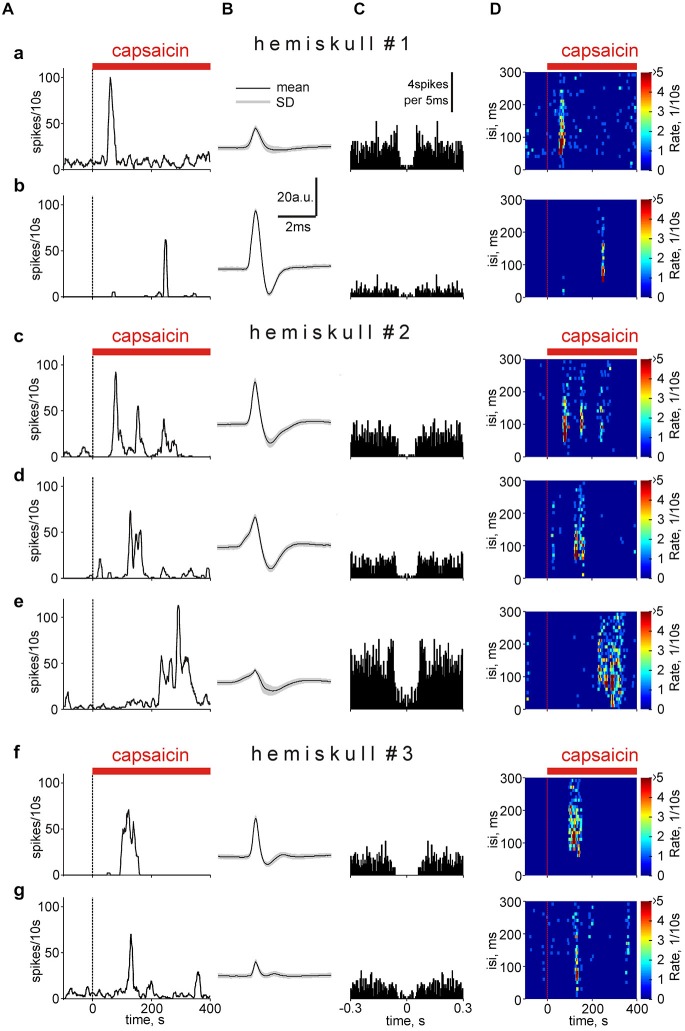
**Temporal analysis of capsaicin effect in different spike clusters. (A)** The variable time-course of capsaicin effect in different clusters in the *nervus spinosus* grouped from three different experiments (hemiskulls 1–3) on the basis of the response latency and persistence. Notice the presence of early transient responder **(a)**, late transient responders **(b)**, persistent responders **(c)** and late persistent responders **(e)**. The width of the window was 500 s. **(B)** Average spike shapes for presented clusters: **(a)** *n* = 826; **(b)** *n* = 88; **(c)** *n* = 606; **(d)** *n* = 451; **(e)** *n* = 1080; **(f)** *n* = 270; **(g)** *n* = 587 events within the cluster. **(C)** Autocorrelogramms for corresponding spike clusters (5 ms bin). **(D)** Color spectrograms illustrating the temporal profiles of interspike intervals for corresponding clusters.

**Figure 9:**
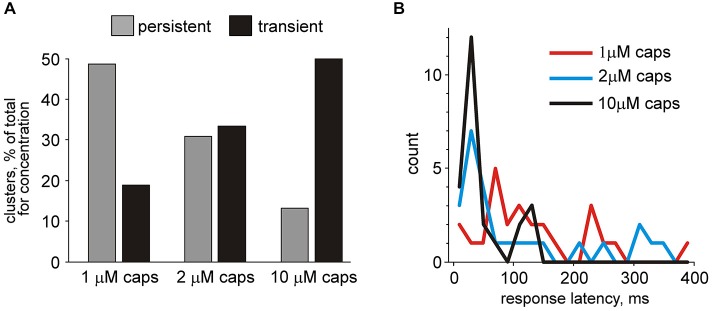
**Quantification of time profiles of clusters in** ***nervus spinosus*** **differently responding to capsaicin. (A)** Histograms showing persistence of responses of all clusters in *nervus spinosus* to application of three different concentrations of capsaicin. Notice an increase in the number of transient type responses proportional to agonist concentration. **(B)** Histograms showing the latency of responses capsaicin (74 responding clusters from 23 preparations).

We further compared the time profile of clustered single unit responses to capsaicin within the same nerve. Notably, in some nociceptive neurons the response duration, rather than firing rate is used as a sensory transduction code (Zhang et al., 2004). Interestingly, in the same nerve (hemiskull #1) one cluster showed very short (50 s) latency (early responder) to application of 2 μM capsaicin (Figure 8Aa) whereas the other cluster (Figure 8Ab) responded with a latency of 240 s (late responder). In both cases the duration of responses was short (around 20 s) indicating a transient type response. In a different meningeal preparation (hemiskull #2), there was also a temporal shift in the onset of long-lasting (persistent) responses between three different clusters (compare Figures 8Ac,d,e). Such a persistent response to capsaicin likely reflected a slowly desensitizing nature of TRPV1 receptors in some terminal branches of the meningeal nerve. There were also intermediate-type responses with similar latency but with differential persistence (Figures 8Af,g). Quantification of these remarkable varieties in the time profile of the responder clusters is shown below in Figure 9.

Figure 8B shows the respective spike shapes for these clusters. Application of the autocorrelation function (Figure 8C) indicated that these clusters mainly reflected activity of single fibers (for a detailed explanation see the *The nature of the cluster of spikes and new details of capsaicin action* in the “Discussion” Section). Figure 8D with color maps demonstrates that for some of these clusters the frequencies were high enough (as in Figure 7) to provide temporal summation of excitatory postsynaptic potentials (EPSPs) at the brainstem and spinal cord level.

Next we overviewed the temporal pattern of responses to the TRPV1 agonist in all capsaicin-responding units. Among 74 capsaicin responders, 35 units (“persistent responders”) showed an elevated firing rate in the presence of capsaicin that lasted for more than the median value of 170 s up to 332 ± 21 s long. Others (39 clusters) responded with short transient changes (70 ± 7 s). Figure 9A shows the percentage of transient and persistent responders for all three concentrations of capsaicin. Notably, there was an increase in the number of transient type responses proportional to agonist concentration. Figure 9B shows that differences in the latent period of the cluster appearance activated by 1, 2 or 10 μM capsaicin. Interestingly, that for all three concentrations we observed an essential number of late responders represented as a “tail” of the histograms. As mentioned above both early and late responses could co-exist within the same meningeal nerve preparation. Altogether these data pointed to a high diversity in the temporal patterns of capsaicin evoked firing in nociceptive fibers of the *nervus spinosus*. Persistent activity in certain fibers likely reflecting the slow rate of TRPV1 receptor desensitization may have important functional effects both on continuous arrival of nociceptive inputs to the brainstem and spinal cord and provides conditions for the slow release of the migraine mediator CGRP from TRPV1 expressing peptidergic neurons.

## Discussion

Using the preparation of the rat skull with preserved innervations of *dura mater*, in this study we characterized spontaneous activity and chemical sensitivity of meningeal nerves relevant to the origin of migraine pain. The main findings of the present study are: (i) variable contribution of individual fibers (tonically active and almost silent) to ongoing firing in control and during activation of nociceptors by the TRPV1 agonist capsaicin (responders vs. non-responders); (ii) almost full block of spontaneous or capsaicin-evoked spikes by TTX; and (iii) two mechanisms contributing to persistent capsaicin-induced activity of the trigeminal nerve involving sustained activity of capsaicin responses in individual fibers.

### Characterization of Preparation and the Recording Mode

Since migraine headache originates from trigeminal nerve terminals in meninges (Goadsby, 2007; Levy, 2010) it is important to have a direct experimental approach to study these structures in live tissues. The isolated hemiskull preparation (De Col et al., 2012; Uebner et al., 2014) represents one of most reliable models to study peripheral neuronal mechanisms of migraine pain. The main advantages of this *ex-vivo* hemiskull preparation are: (i) preservation of the basic morphology of the meningeal tissues including meningeal nerves; (ii) control of drug concentration (concentration-clamp conditions which are critical to study desensitization); and (iii) recording of exclusively peripheral nociceptive traffic, thus excluding spikes which could originate from the somas of neurons in trigeminal ganglion (see Amir and Devor, 1996; Thalakoti et al., 2007). Previous studies of meningeal nociception either in the hemiskull (De Col et al., 2012; Uebner et al., 2014) or *in vivo* (Zhao and Levy, 2014) were mainly dealing with the mechanical activation of meningeal nerves. However, typical nociceptors have a high threshold for mechanical activation (Costigan et al., 2009). Thus, in the current study we mainly focused on the chemical sensitivity of nociceptors of meningeal nerves by analyzing the effect of the algogen capsaicin on nociceptive firing in concentration clamp conditions and by applying the clustering approach. The *nervus spinosus* (part of the V3 branch of the trigeminal nerve) used for our recordings mainly innervates the region of the MMA (Figure 1A) known to be one of the key contributors to migraine pain (Uebner et al., 2014). As an increase in activity of intracranial sensory nerve fibers should provide nociceptive effect (Messlinger, 2009), the afferent traffic which we analyzed in this project is highly relevant to understanding the basic mechanisms of headache in migraine.

Notably, the spikes recorded in the current study, despite their high variability in amplitude, all had a short duration, a characteristic of single fiber action potentials, which makes them different from the compound action potential typically recorded after mechanical activation (De Col et al., 2012; Uebner et al., 2014).

We also found that almost all spikes were blocked by the typical sodium channel blocker TTX. This indicates that the relative contributions of TTX-sensitive and resistant sodium channels to the spike generation in the peripheral nerve differs from that in the somas of sensory neurons (Baker and Waxman, 2012), a traditional model used to study sodium channel pharmacology. Our findings suggest that the selective blockers of sodium channels may have a clinical impact in the treatment of trigeminal pain and headache at peripheral site once a subunit specific profile of these channels in the different fibers of trigeminal nerve in meninges is clearly established. This approach could act in concert with already used sodium channel antagonists destined to reduce cortical excitability in migraine (Chiossi et al., 2014).

### The Nature of the Cluster of Spikes and New Details of Capsaicin Action

The most interesting data were obtained in this project after separating and grouping all spikes with the clustering approach, which was used for the first time with this preparation. First, we found that there are some tonically active fibers (with frequency up to 5 spikes per second) and essentially silent clusters (with frequency around 0 spikes per second). These silent clusters, however, could be highly responsive to capsaicin. Our novel clustering approach evaluated, within the same *in situ* preparation, the presence of “responder” and “non-responder” units. This is consistent with our results obtained from isolated sensory neurons where analogous responding and non-responding to capsaicin neurons were found. Interestingly, in contrast to “responders”, “non-responders” reduced their activity during capsaicin application. Further analysis allowed the identification of spike type with transient (desensitizing) response and long-lasting (slowly desensitizing) clusters.

The typical number of clusters detected with this method varied from 3–10 although the number of fibers in this nerve is much higher (Figure 1B). The question arises whether each cluster corresponds to the group of fibers or it reflects the activity of a single fiber. One approach to solve this issue is based on correlation between neighboring spikes (Johnston and Wu, 1995; Fee et al., 1996) within one cluster (autocorrelation function). The main assumption was that on the autocorrelation function for a single fiber there should be an empty space around zero, about 40 ms (−20, +20 ms) long, as expected from shortest intrerspikes intervals (~20–30 ms) obtained from our analysis of capsaicin-induced firing of a single trigeminal neuron (Figure 6D). In fact, this predicted empty space was indeed found in autocorrelation functions (Figure 8C). In general, 24/114 clusters suggested that their activity reflected a single fiber firing (see for example Figures 8Cc,F). In some clusters (19/114) the low activity was not sufficient to make a definite conclusion whereas in other clusters observed activity likely resulted from the firing of more than one fiber (Figure 8Ce). Interestingly, the latter had similar amplitudes and temporal characters perhaps forming groups of units with remarkably standard functional properties. Whereas the specificity of our methodological approach did not allow us to distinguish between C- and A-fibers, immunostaining used here suggested that up to 30% of myelinated fibers express TRPV1 receptors and may contribute to responses to capsaicin.

Whereas higher resolution techniques are needed to address all these issues in more detail, the current evidence suggests that at least some clusters reflect the activity of single fibers. Nevertheless, there is a remarkable match between the fraction of capsaicin-sensitive clusters (65% of responders in current study) and our previously published data (Simonetti et al., 2006) on the fraction of isolated capsaicin sensitive trigeminal neurons (60%). The latter supports the view that our cluster analysis is efficient enough to detect the neurochemical profile of nerve fibers within this key meningeal nerve.

### Pathophysiological Implications

Despite the high prevalence of migraine, the neuronal mechanisms of this disorder are little understood. The neurochemical mechanisms of nociceptive firing in the meningeal nerve endings represent an attractive but still unsolved issue important for understanding the neurobiology of migraine and essential for the development of new evidence-based treatments at the origin of pain. The focus on little studied peripheral signaling is especially important because recent data indicated distinct terminal and cell body mechanisms involved in pain (Ferrari et al., 2015).

Our data indicate that more than half of the active clusters of meningeal spikes respond to the classical algogen capsaicin indicating a high functional pool of TRPV1 positive fibers in the meningeal *nervus spinosus* innervating region of MMA, a receptive field for migraine pain. We also found that the long-lasting integrative activity evoked by the TRPV1 agonist capsaicin in the whole nerve could be supported by two mechanisms: (i) asynchronous involvement of early and late responding clusters; and (ii) by the presence of clusters with intrinsically persistently active fibers (probably, due to slow rate of desensitization of TRPV1 receptors in these fibers). A co-existence of TRPV1 receptors with slow and fast kinetics was described earlier in isolated sensory neurons (Akopian et al., 2007; Storti et al., 2015) and confirmed in this study in isolated trigeminal neurons. However, expression of these kinetically distinct TRPV1 receptors in peripheral nerves was not previously shown and their functional implications remained unclear. We show here that such kinetic dualism exemplified by the presence of transient vs. long lasting capsaicin-induced clusters takes place also in meningeal nerves. Long lasting activity within meningeal nerves may have two functional implications. On one hand, repetitive firing provides a continuous traffic of action potentials to the presynaptic part of central processes of trigeminal neurons to activate second order nociceptive neurons in the brainstem (Kim et al., 2014). The appearance of spikes with a >10 Hz frequency within the very same cluster suggests that such traffic is high enough to provide postsynaptic temporal summation of nociceptive excitation at the brainstem and spinal cord level. Notably, repetitive flow of excitation can not only enhance the synaptic efficacy but can eventually induce activity-dependent plasticity of brainstem and spinal networks, underlying central sensitization known to be induced by peripheral application of capsaicin (LaMotte et al., 1992). On the other hand, at the periphery, the prolonged activity of trigeminal nerve terminals can support the slow release of the key migraine mediator CGRP, sensitizing trigeminal neurons in an unusually slow manner (Fabbretti et al., 2006; Simonetti et al., 2008). It is worth noting that while used as a classical tool to study chemical activation of nociceptors, capsaicin is an *exogenous* agonist of TRPV1 receptors. However, there are other lipid messengers suggested as *endogenous* agonists of TRPV1 receptors (Morales-Lázaro et al., 2013). Such lipid messengers (endovanilloids) are probably released in migraine with aura during cortical spreading depression (CSD) or in migraine without aura after stress induced mast cell degranulation in meninges (Levy, 2010). These diffusible (due to lipid nature) messengers could act, like capsaicin, in the same spatial and temporal manner to provide massive and persistent activation of meningeal nerves as shown in the current study. Whereas the role of the putative TRPV1 receptors in migraine pain is controversial (Dussor et al., 2014) TRPV1 receptors may act in concert with transient receptor potential ankyrin 1 (TRPA1) receptors whose role in migraine is currently recognized (Edelmayer et al., 2012; Benemei et al., 2014). Co-activation of TRPV1 and TRPA1 receptors could produce large synergy in promotion of CGRP release (Shatillo et al., 2013). In addition to concerted contribution to CGRP release (Shatillo et al., 2013), TRPV1 and TRPA1 receptors making common functional units (Salas et al., 2009) in the same population of trigeminal neurons can also together contribute to migraine pain.

To sum up, the cluster spike analysis unmasking multi-component and temporally distinct profile of nociceptive traffic, activated in this study with the classical pain trigger capsaicin, could be a novel approach to study various types of trigeminal (including migraine) pain.

## Conflict of Interest Statement

The authors declare that the research was conducted in the absence of any commercial or financial relationships that could be construed as a potential conflict of interest.
